# The Impact of Natural Elements on Environmental Comfort in the Iranian-Islamic Historical City of Isfahan

**DOI:** 10.3390/ijerph17165776

**Published:** 2020-08-10

**Authors:** Kyoumars Habibi, Seyedeh Maryam Hoseini, Majid Dehshti, Mojtaba Khanian, Amir Mosavi

**Affiliations:** 1 Department of Urban Planning and Design, Faculty of Art and Architecture, University of Kurdistan, Sanandaj 6617715175, Iran; 2 Department of Urban Planning and Design, Faculty of Art and Architecture, Iran University of Science and Technology, Tehran 1684613114, Iran; hoseini.iust@yahoo.com; 3 Department of Urban Planning and Design, Faculty of Art and Architecture, Shahab Danesh University, Qom 3711687764, Iran; deheshti_m@yahoo.com; 4 Young Researchers and Elite Club, Hamedan Branch, Islamic Azad University, Hamedan 6518764811, Iran; khanian.mojtaba@gmail.com; 5 Faculty of Civil Engineering, Technische Universität Dresden, 01069 Dresden, Germany; 6 Institute of Research and Development, Duy Tan University, Da Nang 550000, Vietnam; 7 Department of Informatics, J. Selye University, 94501 Komarno, Slovakia

**Keywords:** urban microclimate, thermal comfort, cooling effect, urban hazard, natural elements, climate change, sustainable urban development, urban heat island, smart city, sustainable city, eco-friendly, global warning, natural hazards, built environment

## Abstract

Cities directly influence microclimates. As the urbanization expands, and the green spaces diminish, the heat islands begin to emerge. An old technique used during the past centuries—in both hot and dry climates of the central cities of Iran—was the moderation of microclimates via water and plants. With a diachronic approach to the study of the historical Chahar Bagh Street in Isfahan, this paper investigates the impact of the structural changes on its microclimate in three different scenarios, i.e., the street with its features during the Safavid Era (from 1501 to 1736); the street in its current status; and finally a probable critical condition resulting from complete elimination of natural elements from the environment. The mixed strategy used in this study relies on logical reasoning and software-assisted evaluation for comparing the three scenarios. The predicted mean vote (PMV) model was used for measuring thermal comfort. The results indicate that the evaluated comfort-providing area in the Safavid scenario is 7–17 times more favorable than the others. Moreover, the temperature in the contemporary era was found to be 1.5 degrees Celsius cooler than that of the critical status scenario.

## 1. Introduction

Sustainable urban development is of utmost importance for the well-being of citizens in the era of rapid urbanization. Significance of the livelihood of the cities of the future, within the context of climate change and global warming, has been emphasized by the General Assembly of the United Nations (UN) which adopts the resolution “Transforming our world: the 2030 Agenda for Sustainable Development” (United Nations 2015) [1]. The vision presented for “transforming our world for the better” is based on five values, or, the so-called “5 Ps”, i.e., people, planet, prosperity, peace, and partnership, to be realized through a set of Sustainable Development Goals (SDGs) [2]. Thus, moving towards more eco-friendly cities and environmentally conscious urban planning have been widely adopted by local governments and research centers.

Isfahan’s historical school of architecture emphasizes the sustainable integration of the city with the surrounding environment and complete harmony with nature. This approach conforms to the three principles of “impact on the environment”, “relation with nature”, and “understanding natural flows” [3]. However, many of Isfahan’s gardens have been destroyed because of urbanization trends during recent decades. Gardens and green spaces are often the first victims of the industrialization process and migration of rural residents to urban areas. A glance over the maps of Isfahan during different eras clearly shows the diminishment of the urban vegetation, especially in the central area.

**Figure 1:**
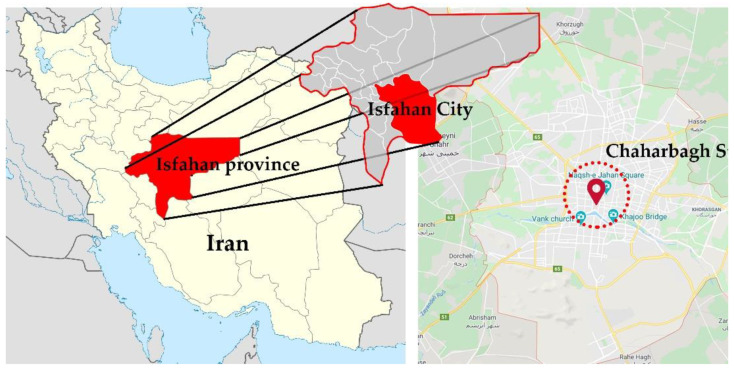
The location of Isfahan City and Chahar Bagh Street.

Urban vegetation improves the quality of life and can increase comfort levels by establishing a relationship between city dwellers and nature. The study of the temperature of various surfaces shows that urban parks have a cooling effect on their surroundings based on their dimensions [4]. With this fact in mind, the three indices of temperature, relative humidity, and thermal comfort were analyzed via the study of the old maps of Chahar Bagh Street and Niasarm Madi in Isfahan City in three different scenarios to determine the impact of green space on its surrounding microclimate. Figure 1 shows the location of Isfahan City and Chahar Bagh Street. The first scenario was the condition of the street in the Safavid Era; the second scenario was the current condition of the street; the final scenario was a probable critical condition that can emerge if all of the trees of the site are cut down, and the water canal dries up. The main goal of this research was to study the impact of the gradual removal of vegetation of a specific site on its surrounding microclimate to highlight the importance of conservation of plants and the significance of the functions of green spaces in cities via reliably obtained statistics.

Studying the impact of the city on its surrounding temperature is very significant. The temperature rise resulting from urbanization has been measured to be 1–3 degrees Celsius. However, this difference can reach up to 10 degrees in certain sustained climatic conditions. In some cities, the share of urbanization in the earth’s warming is more than 80%, which makes it the determinant factor [5]. Given the direct effect of greenery on the indices of microclimate, the study aimed to find the relationship between the area of green spaces and the area of urban space, which falls within the range of thermal comfort. Climatic data are the main input of meteorological software for determining microclimatic specifications. Since no climatic data on the Safavid Era is available, it is not possible to accurately reconstruct the microclimatic condition of that historical period. Therefore, this study attempted to reconstruct an expanse of four hectares of the public space of the contemporary era with the physical structure of this space during the Safavid Era. The results represented the features of Chahar Bagh Pass with the assumption that no substantial change has occurred in the street and the adjacent blocks until the present.

This street has undergone many changes in terms of the use of the surrounding blocks, the materials used for construction of the floor and walls and the green areas. In 2018, when Chahar Bagh Street was pedestrianized and became inaccessible to cars, some of the altered sections, such as the flooring and some parts of the walls returned to their original state, and this greatly contributed to revitalization of the space. Increasing sociability and creating a quality public space requires use of trees, water, and proper materials for floors and walls. Absent suitable microclimatic conditions, it is not possible to create an attractive and lively atmosphere, even without the presence of the cars. This study examines the necessity of maintaining vegetation as a passive method for moderating the temperature of outdoor spaces. The street trees and private urban gardens are an important part of the ecosystem. The purpose of this study is to preserve them.

Consequently, our research is designed to contribute to the following SDGs [1]. Goal 3; ensure healthy lives and promote well-being for all, through creating a suitable microclimate for the vitality of the streets and walkability. Goal 6; ensure availability and sustainable management of water and sanitation for all, through the conservation of water resources (Madi and small ponds) and its importance in improving microclimatic conditions. Goal 7; ensure access to affordable, reliable, sustainable, and modern energy for all though use passive methods to create a suitable microclimate. Goal 11; make cities and human settlements inclusive, safe, resilient, and sustainable. Goal 13; take urgent action to combat climate change and its impacts. Goal 15; Protect, restore, and promote sustainable use of terrestrial ecosystems, sustainably manage forests, combat desertification, and halt and reverse land degradation and halt biodiversity loss.

## 2. Literature Review

One of the first studies on urban climate was conducted by Luke Howard (1818) in London [6]. Considering the significance of urban climatology and its application in urban planning and design, the act of creating and using city maps for analyzing and assessing environmental information is now a common practice. The phrase “urban climate map” was first proposed by German scholar Knoch in the 1950s [7]. These maps are often used for displaying climatic phenomena and related problems [8].

Ye Hai developed and expanded on the concept of thermalscape and categorized it into two classes of natural and artificial. Natural thermalscape refers to the utilization of the earth’s natural features for modification of temperature. Artificial thermalscape, on the other hand, refers to targeted designing such as building green spaces in three dimensions, adding humidity to the air, shading, etc. to create a favorable microclimate [8]. The technique used in Chahar Bagh is a combination of using the natural features of greenery and increasing the local humidity via the addition of water pools and canals along the street. The role that urban ecology plays for regulating the surrounding environment falls under the rubric of passive techniques for improving urban quality of life.

Ecosystems play an important role in the comfort level of their residents; the benefits, however, vary based on the extent and manner of utilizing them. These benefits are often referred to as “ecosystem services”, privileges that humans receive by being in proximity to ecosystems [9]. Many studies have assessed ecosystem services in various urban green spaces such as streetside trees, public parks, private gardens, etc. (Derkzen et al., 2015; Nowak, 1993; Strohbach and Haase, 2012; Sutton Anderson, 2016), as well as management of green spaces (Lilly et al., 2015; Qian et al., 2010) and the effects of various types of vegetation [9]. Most of these studies have focused on the services provided by ecosystems for their surrounding environments, such as carbon footprint or the biodiversity resulting from the richness of green spaces, but none have diachronically researched the green spaces of a specific site and the changes in the surrounding microclimate.

Planting trees is one of the most effective measures that can be taken to reduce thermal tensions in urban areas. The cooling effect triggered by large-scale tree plantation is referred to as park cool island (PCI)—a phenomenon created by green spaces via two mechanisms, which complement one another: shading and transpiration [10]. The reported findings of a wide range of related studies with different temporal and spatial scales lend support to the above-said statement. In addition to the quantitative investigation of PCI and its share in thermal comfort, some studies have researched people’s subjective perception of thermal comfort, and the results validate the physical findings of other studies to a large extent.

Previous studies of urban trees were mostly based on empirical evidence using field measurements (Shashua-Bar et al., 2011). There are only a few numerical simulations with limited spatial coverage using neighborhood-scale models such as ENVI-met (Middel et al., 2015) [11]. Several applications of ENVI-met have been proposed for urban assessments [12,13], considering vegetation [14]. Fahmy et al. suggested a couple of outdoor and indoor simulations using ENVI-met and DesignBuilder to assess the effect of urban trees in the outdoor environment on indoor thermal comfort [15,16]. Morille et al. applied coupling to calculate building energy consumption, considering outdoor conditions of a street canyon “using the SOLENE thermo-radiative model coupled with the outside airflow computed with the Computational fluid dynamics (CFD) (CFD tool Code Saturne) tool Code Saturne” [17]. In the application of the CFD model, Zhao Jing compared the thermal environment of the street canyon under the three species of vegetation based on the Fluent simulation program [18]. Wang develops a stochastic algorithm to estimate view factors between canyon facets in the presence of shade trees based on the Monte Carlo simulation [19].

Studies based on the street cluster thermal time constant (CTTC) model have been carried out by Shashua-Bara and Hoffman [20]. Honjo [21] applied the turbulence model to study the cooling effect of the green space on the surrounding environment. One particular framework of this kind is the mesoscale Weather Research and Forecasting (WRF) model coupled with urban land surface processes parameterized by urban canopy models (UCM). Recent years have seen continuous development in the coupled WRF-UCM framework by incorporating urban trees [11].

In their study of urban spaces, Kolokotroni et al., (2006) used the artificial neural network model to predict the parameters of urban microclimate. Other researchers have used the energy balance models and the computational fluid dynamics model, and sometimes a combination of both, to analyze urban microclimates [22]. However, these studies have mostly been field or laboratory measurements as reliable simulation software is a rather recent product that became available only in the past few decades. The significance of using simulation software lies with the fact that they allow researchers to assess the shortcomings and impact of a project before its physical implementation. They also make it possible to evaluate different situations simultaneously for the selection of an optimum design.

Simulation software has different function areas and almost all of them are used for simulating energy inside buildings except for ESP-r and EcoCities, which can be used for both indoor and outdoor spaces. Among this software, SOLWEI, UMEP, and ENVI-Met have been specially designed to simulate urban environments. Due to its precedence over (in use since 1994) and comprehensiveness compared to other similar software designed for analysis of urban properties, ENVI-Met was used in this study.

One of the most significant elements in the reconstruction of the historical Iranian city of Isfahan during the Safavid period (late-sixteenth century to late-seventeenth century) was the institution of the Chahar Bagh Avenue and its surrounding gardens. Inextricably attached to the creation of this great public space was the theme of the paradisiacal garden—an enclosure complete with landscaping and planting—tended and watered, which excluded the wilderness.

The garden ultimately resulted in a remarkable synthesis of political views expressed through Safavid architectural, artistic, and urban representations. The Avenue has continued to characterize the urban pattern of Isfahan. Today, it remains the most prominent town axis that continues to retain a dialectical relationship between the now-transformed gardens and the ever-evolving city [23]. Focusing on Chahar Bagh Avenue’s physical configuration, this study traces the effect of vegetation and water in the quality of public space.

Many studies have been done on climate change in historic cities. This includes the development of an outdoor thermal comfort model in urban historical areas as defined by Nasrollahi et al., [24]. Furthermore, Shirani-bidabadi et al. analyzed urban heat island (in Isfahan) through thermal analysis [25]. Vadiati and Kashkooli investigated the role of public squares in new developments and suburbia in historical cities (Isfahan) from the environmental sustainability aspects [26]. Alternatively, Keller et al. looked at adapting the historic town to the contemporary climatic needs [27]. Gandini et al. researched the ADVICE project, based on a multiscale approach for the management of climate change impacts on cultural heritage located in the urban context [28]. Dodman et al. brought together a wide-ranging and detailed body of information that identified and assessed risk, vulnerability, and adaptation to climate change in urban centers, including historic cities [29]. Anthony Bigio looked at the issue of resilience of cities with respect to natural hazards, in particular, related to climate change through a review of the current situation of the more than 200 cities inscribed on the World Heritage List [30].

### 2.1. Isfahan’s Urban Development

As long, broad, and straight passages defined and outlined by streams and trees, streets have a long history in Iranian urban development. A prominent example of Iranian street could be seen in Rey City back in the early Islamic era. Such passage gained a clearer structural identity within the urban constructions ordered by Shah Tahmasp in Qazvin. However, what Shah Abbas built in Isfahan surpassed the initial models in different respects and set a new definition for urban space. By aggregating past experiences and transcending them into new insight, this approach became an integral part of Isfahan’s school of urban development. Although the principle of the merger with nature in the design of streets predates the construction of Chahar Bagh, thoughtful use of water and vegetation for appealing to all senses in urban space was unprecedented before the formation of this street [31]. In Chahar Bagh, the direction of Isfahan’s development and expansion is defined rationally without any heavy-handed interference in the historical structure and spatial organization of the city. As the pivot of the border between the new and the old spatial organization, Chahar Bagh passes over the Zayandeh Rood River and presents a combination of the natural and the man-made, the organic and the rational, orderliness and disorderliness, etc. [32].

Isfahan’s historical school of architecture follows certain tenets, which are all based on global grammar despite having various local accents. This grammar has consisted of four materialistic orders, namely water, earth, air and vegetation, and one spiritual order. The existence of these four materialistic orders shows that this school emphasizes the merger of the place with the surrounding environment, the natural with the man-made, constructed with the not-constructed, orderliness with disorderliness, etc., in its grammar [33]. In Isfahan’s school, the city merges with the surrounding environment and, therefore, is in total harmony with nature [34].

**Figure 2:**
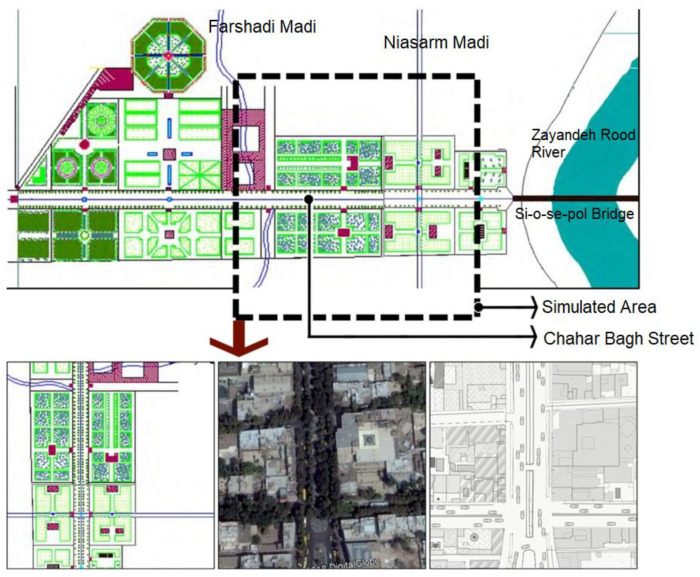
**Up**: Abbasid Chahar Bagh Street during the Safavid Era based on the descriptions in travelogues and the area under study. **Down right**: Critical condition. **Down middle**: Current condition. **Down left**: Chahar Bagh in Safavid Era.

The Abbasid Chahar Bagh Street was constructed during the reign of Shah Abbas the First. This long and broad street that crossed over Allahverdi Khan Bridge and connected the northern area along the Zayandeh Rood River to the expansive colder region of Hezarjarib was one of the main axes of Isfahan’s garden-city project. In an aesthetically pleasing combination with Zayandeh Rood River and Si-o-se-pol Bridge, the Chahar Bagh axis formed a sizable area of various recreational and touristic functions in the structure of the Safavid Isfahan City [35]. Many travelogues mention this street and describe it in detail, which makes it possible to recreate the street with its initial design. Italian explorer Pietro Delavale provides a very detailed description of Chahar Bagh Street during the reign of Shah Abbas I. Figure 2 is the historical map of the Abbasid Chahar Bagh Street during the Safavid Era recreated based on these descriptions and other historical sources.

### 2.2. Theoretical Foundations of the Study

The cities of the central plateau of Iran have always been dealing with thermal comfort problems because of their hot and dry climate. As a result, climatic conditions significantly affect their structures and construction materials. The residents have always tried to create thermally suitable environments in both indoor and outdoor spaces by different methods. Passive techniques, such as the direction and alignment of passages, the dimensions of alleys, construction materials used in building walls and other surfaces, green spaces, the addition of humidity to environments employing water pools and canals, etc., are among these methods. An amalgamation of these techniques could once be seen in the practice of garden building, which has a long history in Iran with Pasargad Garden being the oldest. Iranian garden building reached its heyday during the Safavid Era in Isfahan with the emergence of the concept of garden city [3]. This culmination, manifested in the abundant use of natural elements in urban development for visual landscaping, on the one hand, and improving comfort level, on the other [36,37], appropriately refers to Iranian gardens as “the auspicious marriage of beauty and usefulness”.

**Figure 3:**
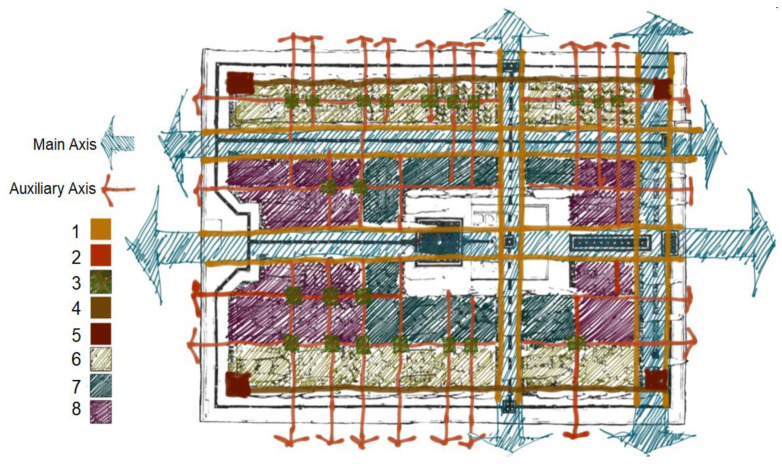
Plantation method based on the location of trees in gardens.

For analysis of the changes in the city’s climate in a four-hectare expanse, an area extending from the beginning of Chahar Bagh Street to a short distance past Niasarm Madi (as indicated in Figure 3) was chosen as the area under study. The reason is that the natural elements of water (in the form of Madi and pools) and greenery (in the form of the treed axis and Safavid gardens) can be found in this area.

Numerous studies have been conducted on thermal environments in human settlements since 1920 and most measurement indices have developed based on temperature and relative humidity. Currently, there are many indices based on body temperature, which can be used for assessment of thermal comfort in a place, including predicted mean vote (PMV), effective temperature (ET), standard effective temperature (SET), the standard effective temperature used in outdoor situations (OUT-SET) and physiological equivalent temperature (PET) [38].

The PMV model, one of the most common of these methods used in this study, was proposed by Fanger in 1972. This model uses the principles of thermal balance for assessing thermal comfort via the mean response of individuals in the sense that if a certain percentage of the individuals in a place feel comfortable, that place falls within the range of thermal comfort [39]. Usually, the four environmental indices of air temperature, mean radiant temperature (MRT), wind speed, and relative humidity and the two personal indices of clothing and metabolism (amount of physical activity) are used for calculation of PMV [40]. According to ISO 7730, the suitable PMV for having comfort during daytime and nighttime is ±0.5 and ±1, respectively [41]. Positive numbers indicate the need for cooling and negative ones indicate the need for heating, both relative to the value of the number. Leonardo software determines areas that have thermal comfort based on the PMV index.

The three indices of temperature, relative humidity, and comfort condition (via the PMV model, which was fully described in the previous paragraph) were measured in this research. The combination of temperature and relative humidity determines the thermal suitability of an environment. Reducing temperature without adding much to humidity has a more notable impact on thermal suitability. In this study, the type of metabolism (amount of physical activity) was decided to be “relaxed walking” and the type of clothing was decided to be suitable for the simulation season (summer). All other temperature information was calculated by the software and the comfort condition was assessed based on the PMV model via coalescing two said indices.

## 3. Methods and Materials

This research was conducted to show the efficiency of historical cities in modifying the microclimate of their public spaces via passive techniques. Considering the significance of the cooling effect of transpiration and the role of tree shades and water in the main indices of microclimate, a digital simulation was used to achieve the set goals. This study had a quantitative approach and relied on logical reasoning and digital modeling. The area under study was a four-hectare expanse of Chahar Bagh Street and the adjacent blocks around Niasarm Madi. The hottest day of the 20-year statistical period between 1998 and 2017 was chosen as the simulated day so that the environmental conditions could be analyzed at their most critical level. The simulation compared and contrasted three different scenarios to analyze the changes in temperature, relative humidity, and airflow, which are the main constituents of thermal comfort. The three scenarios were the historical form of Chahar Bagh Street during the Safavid Era, its contemporary form as a footpath with the existing floor and wall materials, and a critical condition resulting from the removal of natural elements from the urban space.

**Figure 4:**
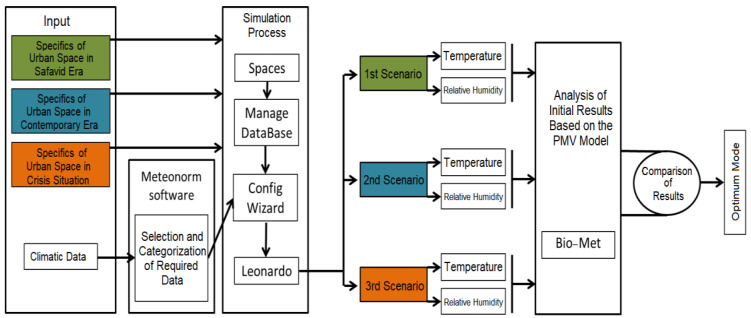
The methodological process of the study.

The required data on the existing urban space and physical structure of the area under study were collected by field measurements and the required climatic data were acquired from the documents provided by Iran Meteorological Organization. The input data used in the simulation software were acquired from Iran Meteorological Organization’s office in Isfahan City. The thermal information collected in the location was used for verification of the software results. This information was obtained on Chahar Bagh Street with the help of a thermometer on 27 January, 2019. First, the simulation was run in ENVI-Met 4. Then, the necessary outputs were obtained with the help of Leonardo software. Bio-Met and the PMV model were used for the analysis of the collected information. Finally, the impact of design on the alteration of microclimatic indices was measured by a comparison of the results. For testing the reliability of these results, air temperature was measured on the 27 January, 2019, and then it was simulated in the software. A comparison between the two cases established the reliability of the research methodology. Figure 4 indicates the methodological process of this study.

This study did not take into account the effect of irrigation of the gardens that have been established on both sides of the street on the microclimate of the space. This was one of the significant limitations of the study. Due to lack of sufficient information about the method and amount of irrigation of these gardens during the Safavid Era, only the effect of the Madi and the pools were analyzed.

The dimension of 60 × 60 × 60 units was used for the simulation in which each unit was equal to 4 m. For increasing the simulation accuracy, five units from each side were omitted and discarded in the results. For determining the simulation day, the average of the hottest day from 1998 to 2017 was obtained. Table 1 show the climatic data. The tree types were different in the first and second scenarios, which has been fully explained in the Figure 3. There was no tree in the third scenario. Table 2 shows the materials used in the floors and walls in detail.

### 3.1. Simulation

The Abbasid Chahar Bagh Street was simulated in three different scenarios as described in Table 2 for displaying the impact of water and greenery on urban microclimates. The first scenario was the original design of the street during the Safavid Era with all the details. The second scenario was the current condition of the street with all the changes that have occurred throughout time. The third scenario was a critical condition in which the street was envisioned as a typical passage with no streetside trees or water canal (Niasarm Madi). Figure 3 indicates the three scenarios.

#### 3.1.1. Simulated Day

For any such simulation, the target day needs to be one with the highest need for temperature adjustment. According to the available statistics, the hottest day of the twenty-year statistical period from 1998 to 2017 was the 13 July 2012. Therefore, according to Table 2, the twenty-year mean of July’s temperature was given to ENVI-Met as input.

#### 3.1.2. Simulation Conditions

Since thermal comfort in urban environments is mostly related to pedestrians, the height was decided to be 1.8 m (average human height) from the surface. The simulation hours were 09:00 a.m., 12:00 a.m. and 02:00 p.m. After the simulation and measurement of temperature and relative humidity, the calculations performed by ENVI-Met were entered into Bio-Met and the final resulted were obtained based on the PMV model. Since the simulated day was decided to be the hottest day of summer, the clothing coefficient was set to 0.57 (trousers and t-shirt) and the activity was set to “walking” [43,44] for assessment of climatic comfort.

#### 3.1.3. Vegetation Type

The trees in the current status scenario are as follows. Due to the changes that were made in the use of the adjacent gardens, trees can be seen only on the street itself. Table 2 shows the number of tree rows.

Choosing the same types of trees that were once planted in the past was very significant for increasing the simulation accuracy. Therefore, historical documents and sources were reviewed for accurate recreation of the street. Travelers who visited Isfahan during the Safavid Era have said that two rows of plane trees outlined the margin of Chahar Bagh Street and almost every travelogue mentions the water canal running through the middle of the street as well as the water pools. These travelogues also describe several water pools in Sardar Azam Mansion [45]. Regarding the trees in the private gardens on both sides of the street and the trees on the street sides, usually, shade-providing trees such as cypress, plane, and willow are planted. Moreover, small gardens in which fruit trees or plants can be gown are built [46]. Overall, in the main streets of that era, cedar, pine, and plane trees were typically planted with an alternating pattern, which was repeated on the opposite side as well in a manner that each tree faced a similar species across the street [47,48].

Ershad Al-Zera’a, authored by Qasem Bin Yousef Abu Nahr Heravi in 1533, explains how gardens were built in the Safavid Era. The planting method along the walls of most gardens conforms to what Heravi describes in his book [47], and also explains how trees were planted back in that era. Table 1 and Figure 3 show the situation and species of the trees planted in Iranian gardens, according to Pirnia. Criteria for Urban Landscape Design, no. 203, was used as the reference for determination of the trees, which are suitable for Isfahan’s climate with an Irano-Turanian geographical distribution [49].

## 4. Results 

### Validation

**Figure 5:**
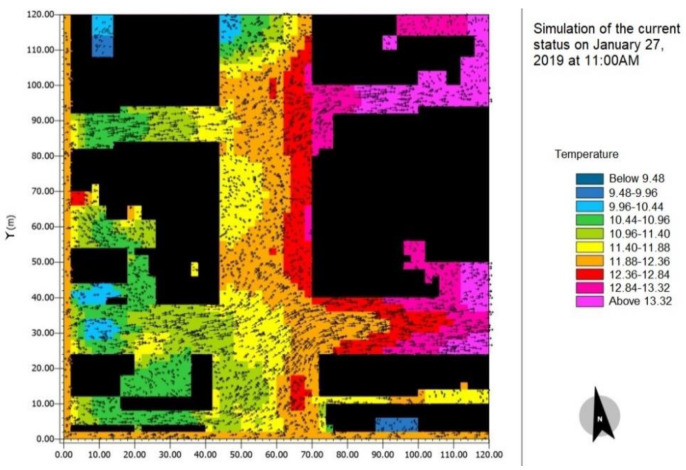
Simulation of 27 January 2019, for validation purposes.

For validation of the software results in Figure 5, a simulation was run for 27 January 2019, in winter condition with the maximum and minimum temperature of 14 and 4 degrees respectively, the relative humidity of 7%, wind speed of 9 km/s, cloud cover of 15%, and wind direction of 260 degrees [50]. The obtained temperature was the same as the physically measured one, and this validated the results.

**Figure 6:**
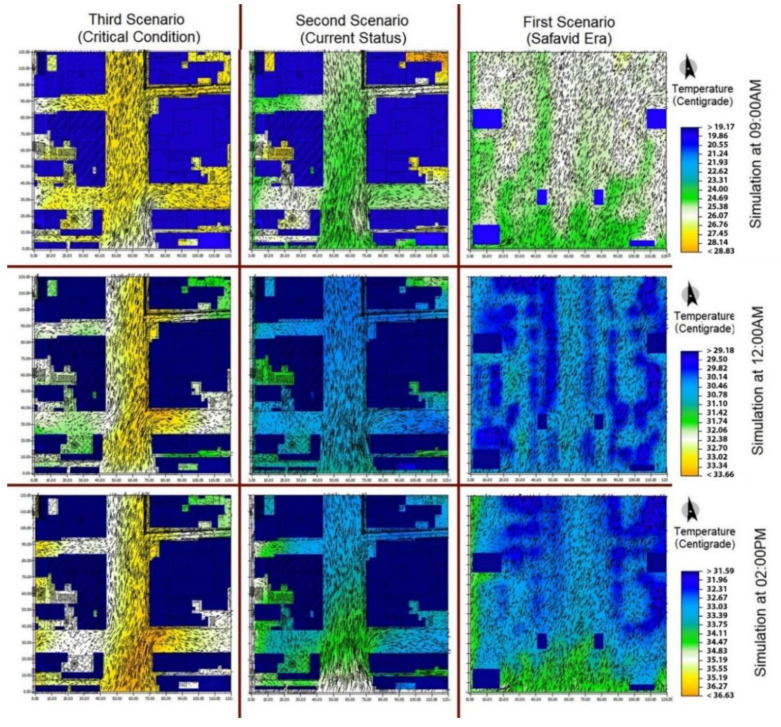
Comparison of temperature in the three scenarios during different hours of the day.

As can be seen in Figure 6, the temperature in the first scenario, especially during the heat peak hours, is notably different from that of the two other scenarios because of the trees. The tree types in the Safavid and current status scenarios have been discussed in detail in the Section 3.1. In the third scenario, the trees were assumed to have been completely removed, except for the early hours of the day during which surfaces were still transferring the coolness of the night to the environment. The temperature of the second scenario was more favorable than that of the first scenario to some extent. In other cases, the temperature of the urban environment in the Safavid scenario was lower by 1–1.5 degrees than that of the current status scenario, and by 2.5–3.5 degrees than that of the critical condition scenario at noon. At 02:00 p.m., the Safavid scenario was cooler than the current status scenario by 2–2.5 degrees and cooler than the critical condition scenario by 3–3.5 degrees. In Figure 6, the thermal condition improves as the colors shift toward the blue side of the spectrum.

**Figure 7:**
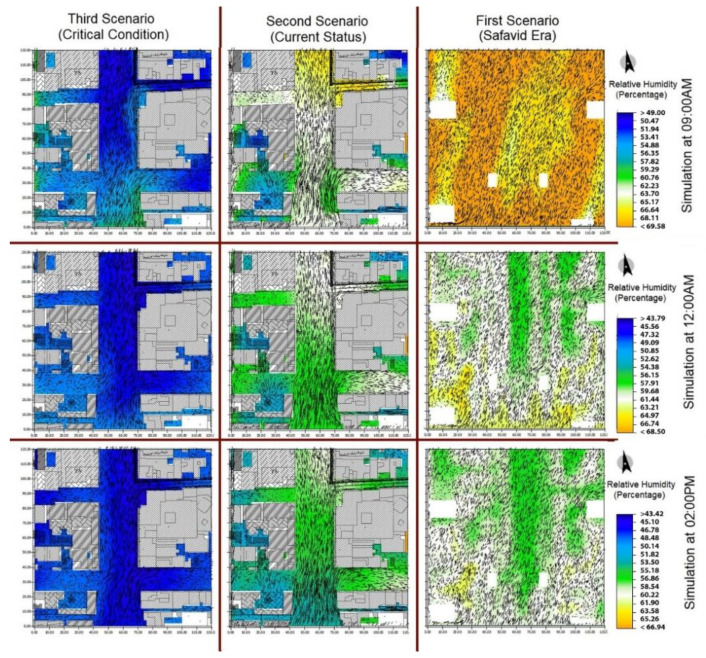
Comparison of relative humidity in the three scenarios during different hours of the day.

**Figure 8:**
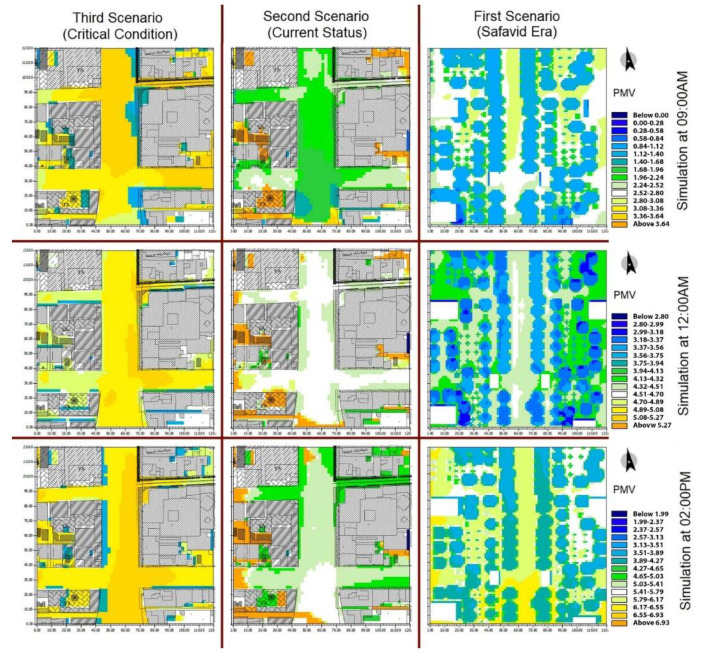
Comparison of thermal comfort in the three scenarios during different hours of the day.

Figure 7 shows relative humidity in the three different scenarios. It increases as the colors shift toward the yellow side of the spectrum. In addition to the trees, the water canal and the pools play an important role in adjusting the air’s relative humidity. As can be seen in Figure 7, relative humidity is very low due to the absence of transpiration and evaporation sources. Relative humidity cannot determine the thermal comfort of a place by itself and it should be taken into consideration along with the temperature. Figure 8 shows the level of thermal comfort based on the indices of airflow, temperature, and relative humidity in the PMV model.

## 5. Discussion

Different combinations of temperature and relative humidity can bring about thermal comfort. For example, when relative humidity exceeds a certain amount, it can cause discomfort; however, the same amount of humidity can create comfort in lower temperatures. Therefore, the temperature and relative humidity obtained from ENVI-Met were inserted into Bio-Met so that the level of thermal comfort in individuals could be measured by the PMV model.

Figure 8 indicates that the closer the value obtained by PMV is to zero, the higher the thermal comfort would be. Positive values show the feeling of hotness and negative ones show the feeling of coldness. Considering that the simulated day was the hottest day of the year with the average maximum temperature of 20 degrees (the most critical thermal condition during the year), it stands to reason that the obtained results do not completely fall within the range of thermal comfort. The goal was to make the most of the existing conditions to create the best possible environmental comfort in the urban space using passive techniques.

The total area of the simulated space was 16,647 m^2^. Based on the PMV level shown in Figure 8, the Safavid scenario had the highest thermal comfort. For more accurate results, however, only the area of the public space was compared between the three scenarios. Although the surrounding gardens and blocks were simulated because of their effect on the results, their area was not calculated in the analysis of the final results. The public space selected for this study consisted of Chahar Bagh Street and the space around the Madi and several alleys, which can be clearly seen in Figure 2.

**Figure 9:**
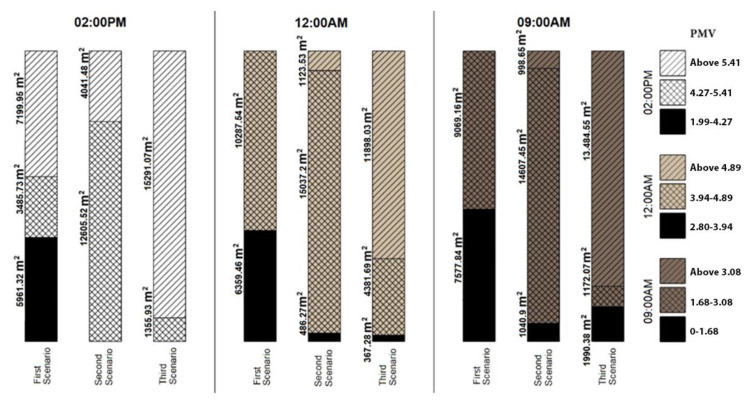
Comparison of the area of the urban environment concerning the predicted mean vote (PMV) classification.

Figure 9 shows this area in the three conditions. At 09:00 a.m. and 12:00 a.m. of the first scenario, the condition is very favorable and no temperature adjustment is required. At 02:00 p.m., however, the rate drops in some sections, which is understandable considering the temperature of the simulated day, which was 40.92 degrees. A comparison between the second and the third scenarios clearly shows the impact of the trees and the Madi on the environment. Although the number of trees and gardens still existing in the area is much smaller than that of the Safavid Era, the impact is significant. In the third scenario, thermal comfort is very unfavorable at all times. The absence of thermal comfort and inappropriate urban landscape resulting from the removal of vegetation from the area would significantly reduce the presence of people in the designated space.

The chart of 09:00 a.m. simulation shows the impact of the rigid surfaces with high heat capacity on the environment’s temperature. This is why the third scenario has more tolerable thermal conditions during the early hours of the day before sunlight intensifies. The impact of vegetation via shading and transpiration becomes tangible throughout the day. In the first scenario, thermal comfort at 09:00 a.m. and 12:00 a.m. was seven times and thirteen times higher than that of the second scenario respectively. Similarly, thermal comfort in the first scenario at 09:00 a.m. and 12:00 a.m. was four times and seventeen times higher than that of the third scenario. At 02:00 p.m., an area of 5960.32 m^2^ of Chahar Bagh was in the range of thermal comfort in the first scenario while the two other scenarios were completely out of the range. Nonetheless, the current status (second scenario) had the far more favorable temperature and relative humidity than the critical situation (third scenario).

While it is not possible to revert the city’s features to their Safavid form, the contemporary condition scenario indicated that streetside trees can be very effective in improving thermal comfort. Due to the high temperature of the simulated day, it was unreasonable to expect the current condition to fall within the range of thermal comfort; however, the current condition was found to be 1–1.5 degrees cooler than the possible critical situation. This temperature difference nudges the existing condition of the area toward thermal comfort during the early and late hours of the day. It needs to be remembered that, so far, the globe’s temperature has risen by almost one degree per century, preservation of this trend over an expanse of four hectares is very significant, and it is possible to notably improve urban thermal comfort by scaling up and benefiting from the techniques used for that purpose.

## 6. Conclusions

Iranian-Islamic historical cities and the related architectural elements, such as Naqsh-e Jahan Square as one of the most prominent public spaces of Iran, have always provided their visitors with climatic comfort via their features throughout history. These features have been inspired and built based on local natural characteristics such as wind, water, airflow, sunshine, shading, natural view corridors, etc., and play an important role in the success of urban spaces and improvement of microclimatic conditions. The comparison between the second and third scenarios showed that choosing the right construction materials for building floors and walls (which affect the environment by their different thermal capacity), planting different types of vegetation, changing the amount of humidity, and other passive techniques, can help create more favorable and suitable environments for outdoor activities so that people spend more time in such spaces. This is one of the strengths of environmental design in Iranian historical desert cities, especially because people’s behavior in open urban spaces is regulated by environmental conditions. In Chahar Bagh Street, small commercial activities, good access as well as identity and historical elements are among the population attraction factors.

However, due to the hot climate of the region, these factors are susceptible to losing their function in case thermal comfort is not provided. The liveliness of Chahar Bagh Street can be guaranteed only by the operation of the collection of all these factors. Any place has its forte and is unique to itself. The presence of water and vegetation in Chahar Bagh is one of its key features and this study attempted to indicate the impact of these natural elements if they are used efficiently to the extent possible. The first step in designing/redesigning a public space is identifying these elements within the scope of the area and making positive changes in the environment with minimum effort and maximum effect. It should be noted that the dimensions of any green space should be determined in accordance with the public space. If a green space is too small, it may not have any tangible effect [51]. The most important advantage of passive design, which was the focus of this study, is indeed maximal use of the environment’s potentials without overspending energy and resource. In addition to its impact on public spaces, creating suitable microclimatic conditions can help conserve energy in indoor spaces via making tangible changes in temperature and relative humidity and casting shadows on surfaces. This study was focused on and limited to public spaces. Analysis of temperature changes in private indoor spaces and the impact of vegetation in other seasons, as well the influence of population density on microclimatic elements, merits further research.

It is also worth mentioning that in the expanded area of 4 hectares, the temperature was measured at five points. Thus, it was not feasible to measure relative humidity and MRT simultaneously. Therefore, the data cannot be considered adequate to draw reliable validation. This poses a limitation to the study presented.

## Figures and Tables

**Table 1:** Location and species of trees. (Numbers guide in Figure 3).

No.	Tree Species	Tree Location
1	cedar, pine, shade trees	on the sides of the main streets
2	mulberry, different types of willow	on the sides of auxiliary streets
3	shade or fruit trees such as pear, apricot, mulberry and sycamine	in the intersection of auxiliary streets
4	poplar and aspen along with jujube and silverberry	along garden walls
5	fig	at the corners of gardens
6	fruit trees such as peach, walnut, almond and, on the eastern side, grape	in rear garden plots
7	flowers based on the region’ climate or Medicago sativa	in garden plots near mansions
8	Medicago sativa (a type of alfalfa grown instead of grass)	between garden plots

**Table 2:** Description of three scenarios of Chahar Bagh Street and climatic components.

Scenario	Situation	Niasarm Madi	Gardens	Row of Trees	Middle Trees and Street Margins	Water Path and Ponds	Middle Flooring	Street Flooring	Wall Material
1	the Safavid Era	✓	✓	1	Existing (Margin only)	✓	stone pavement	stone pavement	Yellow brick
2	current status	✓	✘	2	existing (The middle and margin)	✓	stone pavement	stone pavement	brick and glass
3	critical condition	✘	✘	0	✘	✘	Asphalt	Asphalt	Concrete and glass
Climate Data (Average 20 years 1998 to July 2017) [42]									
Relative humidity	Mean Wind Speed			Precipitation		Wind direction (Prevailing)	Mean Daily Temp	Min Air Temp	Max Air Temp
%36	1.79 m/s			0.81 mm		206.19 m/s	30.37 °C	17.8 °C	40.92 °C
